# A Modified Surgical Technique of Fovea-Sparing Internal Limiting Membrane Peeling: Continuous Arc-Shaped Foldback Peeling

**DOI:** 10.1155/2020/3568938

**Published:** 2020-03-23

**Authors:** Zheng-Gao Xie, Qing-Yi He, Jun Zhu, Wei Du, Jun Tong, Fang Chen

**Affiliations:** ^1^Department of Ophthalmology, Nanjing Drum Tower Hospital, The Affiliated Hospital of Nanjing University Medical School, Nanjing 210008, China; ^2^Department of Ophthalmology, Subei People's Hospital Affiliated to Yangzhou University, Yangzhou 225001, China

## Abstract

**Purpose:**

To investigate the efficacy of management of high myopic foveoschisis (MF) with a modified surgical technique of arc-shaped foldback fovea-sparing internal limiting membrane (ILM) peeling.

**Methods:**

A 23-gauge vitrectomy was performed in five patients with high MF. A long strip of ILM was peeled at the temporal side of the central fovea. Next, an ILM forceps was used to grasp the outer side of the ILM flap, and it was moved forward slowly from the outside to the paracentral fovea, followed by folding ILM back in an arc-shaped manner and then removing it. The above operations were repeated, and all ILM flaps were removed from the outside to paracentral fovea until a narrow strip of ILM remained. Finally, the narrow strip of ILM was excised using a vitreous cutter.

**Results:**

At the patients' last visits, the foveoschisis almost disappeared completely and the fovea reattached. The central macular thickness statistically decreased from 399.0 ± 96.33 *μ*m preoperatively to 164.60 ± 34.20 *μ*m postoperatively (*t* = 4.289; *P*=0.013). The preoperative mean logarithm of the minimum angle of resolution best-corrected visual acuity (1.64 ± 0.65) significantly improved to 0.72 ± 0.18 postoperatively (*t* = 3.265, *P*=0.031). The average follow-up time was 11.80 ± 3.35 months (range; 8–16 months).

**Conclusion:**

The arc-shaped foldback fovea-sparing ILM peeling technique for high MF is safe and effective.

## 1. Introduction

High myopic foveoschisis (MF) is a common complication of pathologic high myopia, and its incidence ranges from 9% to 34% [1]. However, MF pathogenesis remains unclear. The condition occurs in highly myopic eyes due to the tractions exerted by the epiretinal membrane or posterior vitreous cortex on the retina, already stretched by the staphyloma. Besides, the inflexibility of the internal limiting membrane (ILM) is considered to be another important factor, which leads to an intraretinal cleavage before the photoreceptors detach from the retinal pigment epithelium. High myopic foveoschisis can be divided into three types: inner foveoschisis, outer foveoschisis, and mixed foveoschisis. The formation of foveoschisis has a serious impact on central vision acuity. In the observations of Gaucher et al. [2] during the entire follow-up period, nine (31%) of 29 of the eyes remained stable, whereas the disorder progressed in the remaining 20 eyes (69%) and eventually required surgical treatment (the mean follow-up time was 31.2 months). Currently, vitrectomy and ILM peeling are combined with gas tamponade, which was reported to be an effective approach in the management of the disease. Nevertheless, a high risk of the postoperative development of full-thickness macular hole (MH) and macular hole retinal detachment (MHRD) was observed after myopic foveoschisis surgery due to complete peeling of the posterior pole ILM off from the fovea. The risks of full-thickness MH after ILM peeling was reportedly 16.7%–20.8% [3, 4], and MHRD was 5.3% (7/131) [1]. The present report describes an effective ILM peeling technique that leaves the epifoveolar tissue in situ and thus prevents the development of a macular hole.

## 2. Methods

Retrospective case series study. We retrospectively observed the charts of five patients with high MF, who underwent 23-gauge pars plana vitrectomy combined with fovea-sparing ILM peeling, in the Department of Ophthalmology, Nanjing Drum Tower Hospital, the Affiliated Hospital of Nanjing University Medical School, between April 2018 and November 2018. All patients met the diagnostic criteria of pathological high myopia: the axial length exceeds 26.5 mm, and the refractive diopter exceeds −6.0. All five patients were noted to have complicated posterior scleral staphyloma by B-type ultrasonography and myopic foveoschisis by optic coherence tomography (OCT), and four of five patients had a foveal detachment. All patients were operated by the same experienced surgeon (Xie ZG). The surgical method was approved by the medical ethics committee of the Nanjing University Medical School. Patients were informed of the purpose of the treatments and examinations and the possible complications. Written informed consent was then obtained from all patients. The detailed surgical procedure is described below.

**Figure 1:**
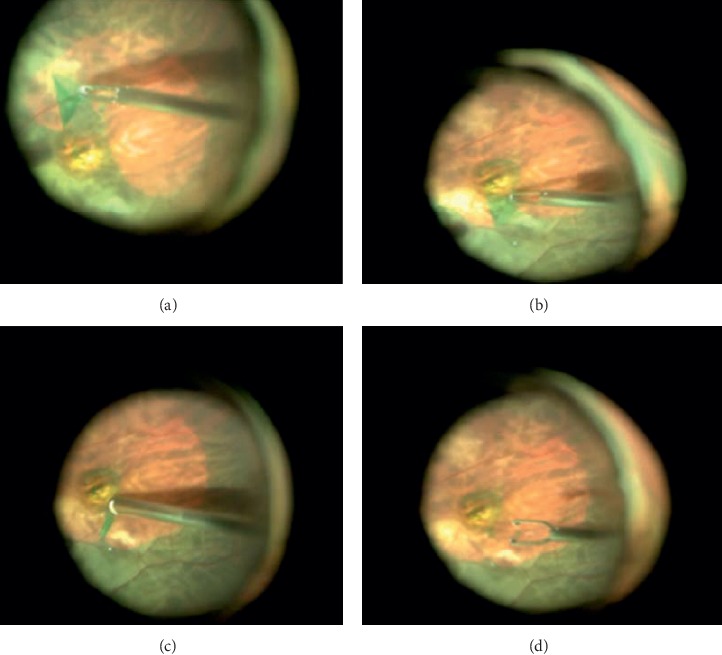
“Arc-shaped foldback” fovea-sparing ILM peeling procedure. (a) After ICG staining, initial ILM tear was performed away from the central fovea at the temporal side, and it was peeled from the outside to the paracentral fovea and then folded back in an arc-shaped manner, followed by removal with special attention not to peel the ILM around the central fovea; (b) ILM flap was made at the superonasal side and peeled toward the paracentral fovea; (c) the narrow strip of ILM was excised with a vitreous cutter. (d) The circular epifoveolar ILM of about 1/3 of the optic disc diameter was preserved.

**Figure 2:**
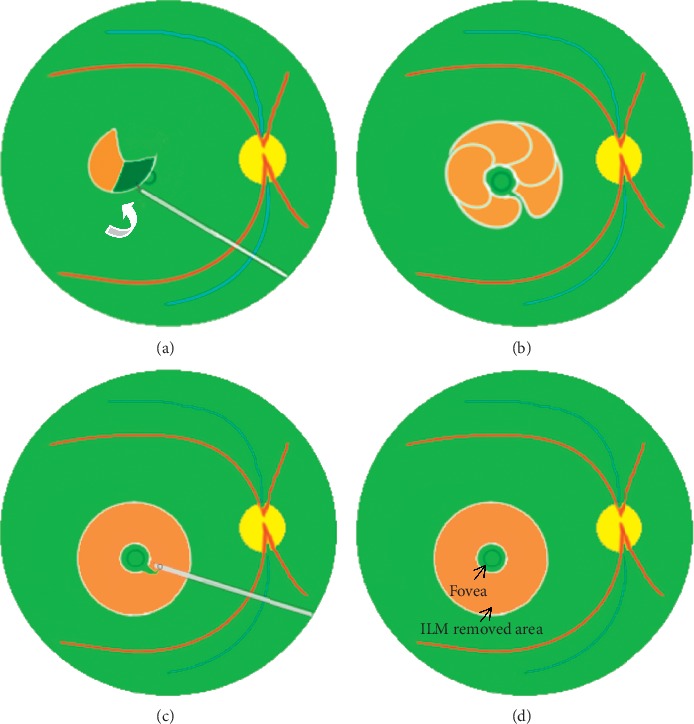
Schematic drawing of “arc-shaped foldback” fovea-sparing ILM peeling. (a) After ICG staining, initial ILM tear was performed away from the central fovea at the temporal side. The outer side of the ILM flap was grasped and moved from the outside to the paracentral fovea (white arrow), caution must be taken not to peel off the central foveal area. (b) The aforementioned operations were repeated, and all the ILM flaps were removed from the outside to paracentral fovea until a narrow strip of ILM remained. (c) The narrow strip of ILM was excised with a vitreous cutter. (d) The circular epifoveolar ILM was preserved.

All patients with high MF were treated with retrobulbar nerve block anesthesia. A standard, 23-gauge 3-port pars plana vitrectomy (Constellation®, Alcon, Fort Worth, TX, USA) was employed in all patients. First, the central vitreous core was removed; triamcinolone acetonide was injected into the vitreous cavity to visualize the vitreous cortex, and posterior vitreous detachment was mechanically induced. Then, the posterior hyaloid membrane was completely removed from the posterior surface of the retina. After complete fluid-air exchange (the intraocular pressure was 35 mmHg) was performed, about 0.5 mL indocyanine green (ICG)-diluted solution (0.25%) was injected into the vitreous cavity to stain the ILM. Approximately 10 seconds later, the ICG solution was entirely removed with a flute, and the balanced salt solution was filled. ILM tear was performed under a contact lens or a wide-field viewing system. A long strip of ILM was peeled at the temporal side of the central fovea with an ILM forceps; an ILM forceps was used to grasp the outer side of the ILM flap, which was next slowly moved forward from the outside to paracentral fovea, and the ILM was folded back in an arc-shaped manner. Shearing forces were used to control the direction to keep the peeling away from the central fovea. The aforementioned operations were repeated, and all the ILM flaps were removed from the outside to paracentral fovea until a narrow strip of ILM remained. Caution should be taken not to totally peel the ILM on the fovea. Finally, the narrow strip of ILM was excised by a high-speed 23-gauge vitreous cutter to prevent peeling the ILM at the foveola. The multiple pieces of ILM peeled away ended up forming an annular region. Circular epifoveolar ILM of about 1/3 of the diameter of the optic disc was preserved (Figures 1 and 2; Supplemental Digital [Supplementary-material supplementary-material-1]). The edge of the epifoveolar ILM was firmly attached to the nerve fiber layer and not warped up. The area of the removed ILM was extended to the temporal side as well as the superior and inferior vascular arcades with an ILM forceps. Finally, an air tamponade was performed. The entire procedure lasted approximately 5–6 minutes. The patients were requested to remain face down after surgery for one week. The macular morphology changes were observed using OCT. The best-corrected visual acuity (BCVA) was transformed to the logarithm of the minimum angle of resolution (LogMAR) for statistic analysis. SAS 11.0 statistic soft package was used. A paired *t*-test was adopted to compare the differences of the central macular thickness (CMT) and BCVA between the preoperation and the postoperation. A level of *P* < 0.05 was considered statistically significant.

## 3. Results

**Figure 3:**
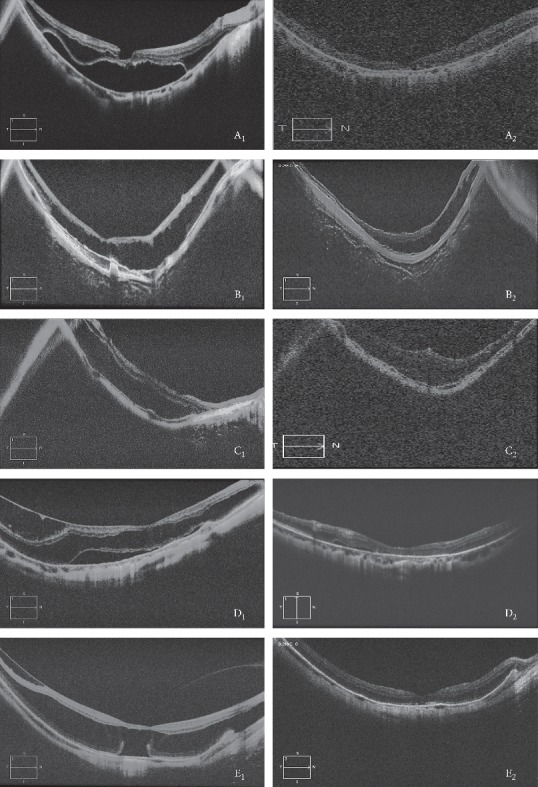
Raster lines comparison report of OCT images (5 lines) before and after surgery. A_1_, the preoperative OCT image of Case 1 showed high myopic foveoschisis and foveal detachment; A_2_, the postoperative OCT image of Case 1 showed that the foveoschisis disappeared and the foveal reattachment; B_1_, the preoperative OCT image of Case 2 showed serious high myopic foveoschisis in the outer layer; B_2_, the postoperative OCT image of Case 2 showed foveoschisis obviously improved; C_1_, the preoperative OCT image of Case 3 showed serious high myopic foveoschisis in the outer layer; C_2_, the postoperative OCT image of Case 3 showed foveoschisis improved; D_1_, the preoperative OCT image of Case 4 showed serious high myopic foveoschisis both in the inner layer and in the outer layer, and detached foveola; D_2_, the postoperative OCT image of Case 4 showed that the foveoschisis disappeared and the foveola reattached nearly; E_1_, the preoperative OCT image of Case 5 showed myopic foveoschisis both in the inner layer and in the outer layer, and detached local foveola; E_2_, the postoperative OCT image of Case 5 showed that the foveoschisis disappeared and the foveola reattached fully.

Five eyes of five patients were enrolled in the study; all five were female and right eyes. The mean age of the patients was 57 ± 9 years old (range; 47–67 years) and the mean duration of onset time 4.80 ± 1.64 months (range; 3–6 months). The average axial length was 30.88 ± 2.60 mm (range; 28.66–34.98 mm) and mean refractive diopter −16.20 ± −1.82 (range; −14.5–19.0 diopters). OCT scans revealed that the foveoschisis was associated with macular anomalies: a premacular structure in three of five eyes, a foveal detachment in four of five eyes. Outer foveoschisis was found in three of five eyes and mixed foveoschisis in two of five eyes. The average follow-up time was 11.80 ± 3.35 months (range; 8–16 months). During the follow-up, vitreous hemorrhage, endophthalmitis, retinal detachment, macular hole, and premacular membrane did not occur. At the patients' last visits, the foveoschisis almost disappeared completely and the fovea reattached. The preoperative CMT was 399.0 ± 96.33 *μ*m (range; 308–563 *μ*m) and the postoperative 164.60 ± 34.20 *μ*m (range; 113–203 *μ*m) (*t* = 4.289; *P*=0.013). In addition, the patients Snellen visual acuity ranged from 20/20,000 to 20/200 preoperatively and from 20/200 to 20/100 postoperatively. The preoperative mean LogMAR BCVA (1.64 ± 0.65) significantly improved to (0.72 ± 0.18) postoperatively (*t* = 3.265, *P*=0.031) (Figure 3) (Table 1).

## 4. Discussion

The current strategies for the treatment of high myopic foveoschisis include external surgery: macular buckle, which is rarely used; internal surgery: pars plana vitrectomy: (1) simple pars plana vitrectomy: intraoperative removal of the posterior vitreous cortex, combined with long-acting gas or silicone oil (SO) tamponade; (2) vitrectomy combined with complete removal of the ILM within or even beyond the vascular arcade; (3) vitrectomy combined with preserving the epi-foveal ILM.

Shimada et al. [4] first proposed fovea-sparing ILM peeling. During the operation, the force direction of the ILM forceps was continuously adjusted, and irregular shape of the ILM flap with an epifoveolar membrane remaining was preliminarily completed. Then, the ILM was carefully trimmed using a vitreous cutter. Ho et al. [5] modified the method of fovea-sparing ILM peeling. In the process of tearing ILM, the ILM was continuously trimmed with microscissors cuts. In this way, the ILM was removed without damaging the central fovea. Repeated microscissors cuts and peeling with an ILM forceps were needed. Jin et al. [6] invented another method of preserving the epi-foveal ILM, in which four circular ILM flaps were peeled with an ILM forceps centered away from the central fovea. After peeling off the residual ILM between the four circular areas, the edge of the residual ILM at the central fovea was trimmed by a vitreous cutter to preserve the epi-foveal ILM. This method may need the application of high operation technology and advanced skills, especially when making four circular ILM flaps. Lee et al. [7] also reported their improved method of fovea-sparing ILM peeling. The ILM was grasped away from the central fovea and peeled off in a circular fashion. Further, the edges of the retained ILM were continuously trimmed with microscissors cuts to adjust the directions of the ILM tearing, so as to achieve the goal of retaining a smaller area of the ILM. This method may also require highly skilled personnel and advanced operation technology and constantly change microscissors cuts and the ILM forceps.

The key points of our modified method were as follows: we peeled a long strip of ILM at the temporal side of the central fovea; an ILM forceps was used to grasp the outer side of the ILM flap, which was slowly moved forward from the outside to paracentral fovea, and the ILM was folded back in an arc-shaped manner, followed by its removal. The main difference of our technique from others is that the fovea-sparing ILM peeling is operated mainly by the use of ILM forceps at one time without repeated trimming; thus, it does not need a repeated change of forceps and vitreous cutter. The diameter of the retained ILM can be estimated during the peeling process. This operation method is similar to the process of capsulorhexis in cataract surgery, but the ILM is not as tough as the anterior capsule. Therefore, the ILM is not peeled accidentally toward the central fovea or the outside, and the force direction could be easily controlled. In other words, the area of the retained ILM could be totally controlled by the surgeon and formed at one time without repeated trimming. Moreover, this technique does not require very high technical skills. An ILM forceps was basically used during the operation, and the vitreous cutter was used at the conclusion of the procedure. Therefore, there was no need to change the instrument repeatedly. Another advantage of this technique was that the edge of epifoveolar ILM was firmly attached to the nerve fiber layer and not warped up.

We employed this surgical procedure in five patients with high MF, and the epi-foveal ILM was successfully preserved in all cases using the arc-shaped foldback fovea-sparing ILM peeling technique. Peeling ILM from the entire macular area and other complications did not occur during the operation. In conclusion, the arc-shaped foldback fovea-sparing ILM peeling technique has the advantages of simple operation, controllable area of the retained ILM, no need to change the instrument repeatedly, and firm attachment of the edge of the epifoveolar ILM to the nerve fiber layer. Therefore, it can be used as an effective alternative surgical treatment method.

## Figures and Tables

**Table 1:** Clinical characteristics of patients.

Case	Sex (F/M)/Age (years)/Eye (R/L)	Time of onset (months)	Axial length (mm)	Refraction status (D)	Pre-op CMT (*μ*m)	Post-op CMT (*μ*m)	Pre-op BCVA	Post-op BCVA	Follow-up time (months)	Secondary macular hole (Y/N)	Secondary premacular membrane (Y/N)
					*t* = 4.289, *P*=0.013		*t* = 3.265, *P*=0.031				
1	F/66/R	6	28.84	−15.50	368	168	20/20,000	20/100	14	N	N
2	F/67/R	6	31.68	−19.00	308	154	20/2,000	20/100	12	N	N
3	F/47/R	6	34.98	−15.00	563	113	20/1,000	20/200	16	N	N
4	F/53/R	3	30.25	−17.00	385	185	20/200	20/100	8	N	N
5	F/53/R	3	28.66	−14.50	371	203	20/200	20/63	9	N	N

F: female; M: male; R: right; D: diopter; Pre-op: preoperation; Post-op: postoperation; CMT: central macular thickness; BCVA: best-corrected visual acuity; Y: yes; N: no.

## Data Availability

The data used to support the findings of this study are available from the corresponding author upon request.

## Supplementary Materials

Supplementary Materials Supplemental Digital Content 1 presents the “Arc-shaped foldback” fovea-sparing ILM peeling procedure.

## References

[1] Huang Y., Huang W., Ng D. S. C., Duan A. (2017). Risk factors for development of macular hole retinal detachment after pars plana vitrectomy for pathologic myopic foveoschisis. *Retina*.

[2] Gaucher D., Haouchine B., Tadayoni R. (2007). Long-term follow-up of high myopic foveoschisis: natural course and surgical outcome. *American Journal of Ophthalmology*.

[3] Panozzo G., Mercanti A. (2007). Vitrectomy for myopic traction maculopathy. *Archives of Ophthalmology*.

[4] Shimada N., Sugamoto Y., Ogawa M., Takase H., Ohno-Matsui K. (2012). Fovea-sparing internal limiting membrane peeling for myopic traction maculopathy. *American Journal of Ophthalmology*.

[5] Ho T. C., Chen M. S., Huang J. S., Shih Y. F, Ho H, Huang Y. H (2012). Foveola nonpeeling technique in internal limiting membrane peeling of myopic foveoschisis surgery. *Retina*.

[6] Jin H., Zhang Q., Zhao P. (2016). Fovea sparing internal limiting membrane peeling using multiple parafoveal curvilinear peels for myopic foveoschisis: technique and outcome. *BMC Ophthalmology*.

[7] Lee C.-L., Wu W.-C., Chen K.-J., Chiu L.-Y., Wu K.-Y., Chang Y.-C. (2017). Modified internal limiting membrane peeling technique (maculorrhexis) for myopic foveoschisis surgery. *Acta Ophthalmologica*.

